# The Effect of Temperature Changes in Vitreoretinal Surgery

**DOI:** 10.1167/tvst.5.1.4

**Published:** 2016-02-09

**Authors:** Mario R. Romano, Vito Romano, Alessandro Mauro, Martina Angi, Ciro Costagliola, Luigi Ambrosone

**Affiliations:** 1 Università degli Studi Federico II, Dipartimento di Neuroscienze e Scienze Riproduttive ed Odontostomatologiche, Naples, Italy; 2 St Paul's Eye Unit, Royal Liverpool University Hospital, Prescot Street, Liverpool, UK; 3 University of Napoli Parthenope, Napoli, Italy; 4 Department of Bioscience and Territory (DIBT), University of Molise, 86090 Pesche (Is), Italy

**Keywords:** temperature, vitreoretinal surgery, surface tension, shear viscosity, perfluorcarbon liquid, silicone oil

## Abstract

**Purpose:**

Recent studies on temperature control in biology and medicine have found the temperature as a new instrument in healthcare. In this manuscript, we reviewed the effects of temperature and its potential role in pars plana vitrectomy. We also examined the relationship between intraocular pressure, viscosity, and temperature in order to determine the best balance to manipulate the tamponades during the surgery.

**Methods:**

A literature review was performed to identify potentially relevant studies on intraocular temperature. Physics equations were applied to explain the described effects of temperature changes on the behavior of the endotamponades commonly used during vitreoretinal surgery. We also generated an operating diagram on the pressure–temperature plane for the values of both vapor–liquid equilibrium and intraocular pressure.

**Results:**

The rapid circulation of fluid in the vitreous cavity reduces the heat produced by the retinal and choroidal surface, bringing the temperature toward room temperature (22°C, deep hypothermia). Temperature increases with endolaser treatment, air infusion, and the presence of silicone oil. The variations in temperature during vitreoretinal surgery are clinically significant, as the rheology of tamponades can be better manipulated by modulating intraocular pressure and temperature.

**Conclusions:**

During vitreoretinal surgery, the intraocular temperature showed rapid and significant fluctuations at different steps of the surgical procedure inside the vitreous cavity. Temperature control can modulate the rheology of tamponades.

**Translational Relevance:**

Intraoperative temperature control can improve neuroprotection during vitreoretinal surgery, induce the vaporization of perfluorcarbon liquid, and change the shear viscosity of silicone oil.

## Introduction

During the last few years, intense research on the biological influence and control of temperature has transformed temperature into a new treatment approach for many human diseases.^1–6^ Alterations of the body temperature of even only few degrees Celsius can have a strong direct or indirect influence on numerous biological processes. For example, morphological and functional changes of membranes and organic molecules induced by a change in temperature of only 2°C can alter the activity of enzymes involved in cellular metabolisms.^7^

Mild to moderate hypothermia (T = 32°C–35°C) can block the cascade of destructive inflammation, prevent blood–aqueous barrier disruption that leads to apoptosis^5^ and local edema, and possibly stimulate the release of protective proteins. In addition, other authors reported that reducing the temperature of plasma could kill pathogens, which was used to disinfect wounds, accelerate wound healing, and induce apoptosis of some cancer cells in vitro.^8^ The application of mild to moderate hypothermia decreases neurological tissue damage caused by ischemia reperfusion, reduces the oxygen metabolic request, and induces the release of protective proteins.^5^ In fact, maintaining body temperature between 32°C to 34°C is one of the treatment options for ischemic cardiac stroke, neurological injury, and neonatal encephalopathy. In addition, mild to moderate hypothermia is currently applied in cardiothoracic or neurosurgical operations to prevent intraoperative strokes.^1,2^ On the other hand, it has been also reported that deep hypothermia (T **<** 32°C) inhibits platelets and reduce the concentration of thromboxane B2, leading to longer clotting times, electrolyte discrepancy, and arrhythmias.^3,6^

In ophthalmology, animal studies in albino rats have demonstrated the protective effects of inducing local hypothermia in vitrectomy under fluctuating intraocular pressure: it reduces stromal edema of the ciliary body and inhibits blood–aqueous barrier disruption.^9^

Clinical studies have been performed by our group and others to measure the intraocular temperature in human eyes undergoing vitrectomy, showing significant fluctuations at different times of the surgical process.^10–12^ Landers et al.^10^ reported that the mean midvitreous and retina temperature in six eyes before vitrectomy with closed infusion line was 34.8°C and 35.2°C, respectively. The authors also reported that the temperature inside the vitreous cavity reached to deep hypothermia and quickly increased after closing the infusion fluid.

Romano et al.^12^ recently investigated the changes of mean vitreous temperature during the vitreoretinal procedures. Temperature was recorded with a 23-gauge thermoprobe in the anterior chamber and on the retinal surface at one time point, whereas the temperature in the vitreous cavity was measured during the entire surgical procedure under fluid infusion and air infusion. At baseline, the temperature was 33.6°C ± 1.4, and it reached to 26.8°C ± 1.0 at the beginning of vitrectomy and 24.8°C ± 0.8 at the end of the surgery under fluid infusion. The temperature fluctuations in different phases of vitreoretinal surgery were statistically significant.

Moreover, variations in intraocular temperature induce changes the surface tension (ST) and shear viscosity (SV) of the endotamponades commonly used during vitreoretinal surgery (i.e., silicone oil [SO] and perfluorcarbon liquid [PFCL]). For example, despite meticulous removal, small amount of PFCL is often found in the vitreous cavity after vitreoretinal surgery due to fluid–air exchange.^13^ The PFCL left on the retina can cause inflammation, emulsification, and sticky oil formation.^14–18^ The viscosity of PCFL plays a basic role when removing PCFL from eyes; therefore, this process can be improved by modulating the intraocular temperature.

Based on the reported biological damage induced by variation of the temperature and on changes induced by temperature on the intraocular compounds, the aim of our study was to describe the best use of the temperature to perform a safer surgical procedures and the best pressure–temperature balance to manage the endotamponades.

## Methods

### Temperature Variations During Pars Plana Vitrectomy

The dynamic changes of temperature are caused by the balance between the inflow and outflow of the fluid in the vitreous cavity. At baseline, the inflow and outflow are both off, whereas both inflow and outflow are on during the vitrectomy, causing the temperature to gradually decrease to deep iatrogenic hypothermia. The temperature increases quickly under air infusion. This could be explained by the fact that gases, such as air, can absorb and conduct the heat energy faster than liquids. The rapid circulation of fluid in the vitreous cavity removes the heat produced by the retinal and choroid surface, reducing the temperature approximately toward room temperature (22°C, deep hypothermia), which is the presumed infusion liquid temperature. Temperature raises again under endolaser treatment, air infusion, and in the presence of SO.

### Temperature and Perfluorocarbon Liquid (PFCL)

PFCL is an intraoperative heavy tamponade commonly used in surgical practice. PFCL has a ST against water of 50 mN/m and a dynamic viscosity of 5.10 mPa·s.^19^ The transparency, low viscosity, and high-density characteristics of PFCL make it widely used to displace subretinal fluid and stabilize detached retina during surgery. Due to the high ST, the PFCL bubble tends to remain round because the interfacial energy creates a net attraction inward.^20^ The Table describes the chemical and physical properties of intraocular compounds used in the vitreoretinal surgery.

**Table:**
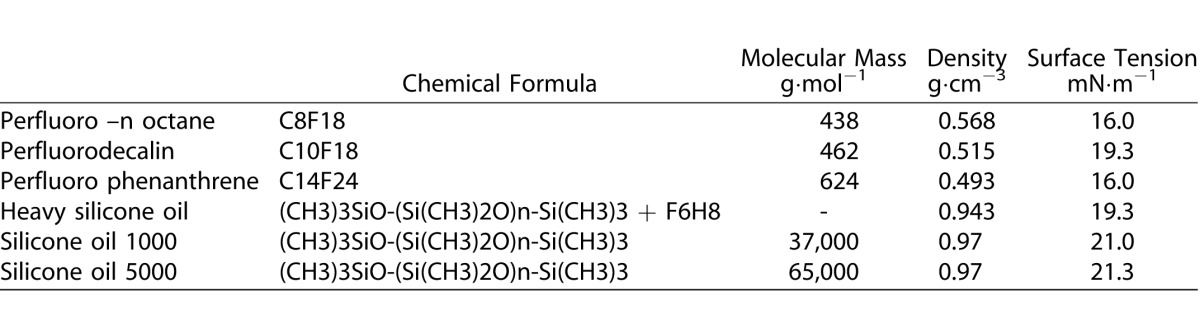
Chemical and Physical Properties of Intraocular Compounds

**Table:**
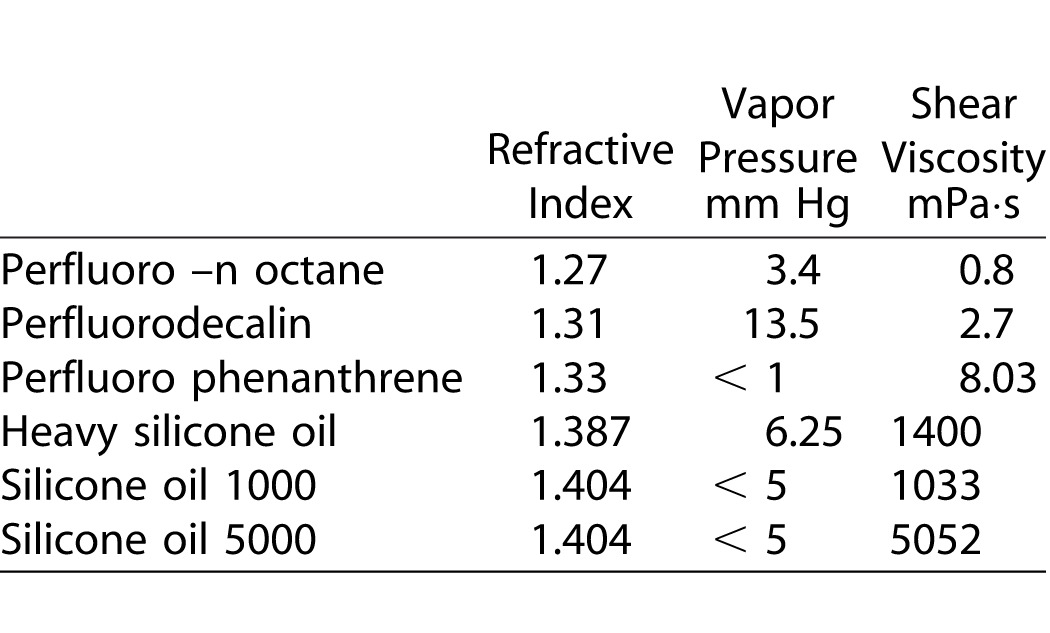
Extended.

From the surface-tension point of view, the order of increasing surface activity is hydrocarbon, silicone, and fluorocarbon. However, from the interfacial tension-against-water point of view, the order of increasing interfacial activity is fluorocarbon, hydrocarbon, and silicone. The silicones do not fit the simple pattern in which reduced surface energy leads to increased hydrophobicity and interfacial tension against water. Due to their backbone flexibility, silicones can adopt various orientations at different interfaces. An orientation involving the interaction between backbone and water phase is thought to generate the relatively low interfacial tension with water.^21^ Why does the terminal CF_3_- (trifluoromethyl) intrinsically exhibit more surface than CH_3_- (methyl)? Van der Waals forces around analogous hydrocarbon and fluorocarbon moieties are nearly identical, therefore, the differences in wettability have to be ascribed to the size difference in the two groups, which results in a lower concentration of attractive centers in the surface in fluorocarbon case.

Correlation of the macroscopic processes of wetting and the adhesion, occurring at interfaces, with the fine structure of tamponades at the molecular level is a fundamental issue in vitrectomy surgery. During the fluid–air exchange, a flow of dry air is in contact with PCL liquid, which evaporates and the air is charged with vapor (evaporated liquid). Determining the amount of fluid removed and its tendency to evaporate is critically important to the success of this operation. The capacity of PFCL molecules to escape into the gas phase is measured by the vapor pressure (i.e., the pressure exerted by the vapor when in equilibrium with its liquid). For a given temperature *T*, there is only one pressure *P*^0^, at which vapor PFCL is in equilibrium with liquid PFCL. The curve separates the liquid region from vapor region, which may be represented in a variety of ways, but none are completely satisfactory.

### Procedures

Based on the literature data, we generated an operating diagram on the pressure–temperature plane for the values of both vapor–liquid equilibrium and IOP.

The Antoine equation:

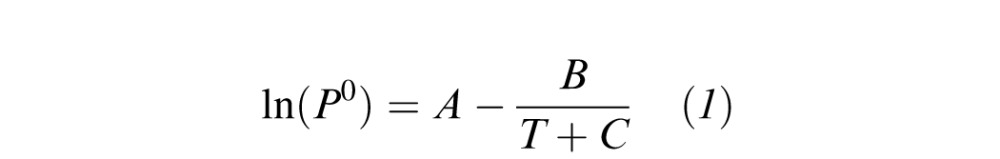
has been found to be absolutely adequate for many liquids, where *A*, *B*, *C* are substance-specific parameters and *T* the absolute temperature. This equation introduces a correction to the commonly used Clausius-Clapeyron equation.

Partial pressure was calculated from the equation of ideal gases:

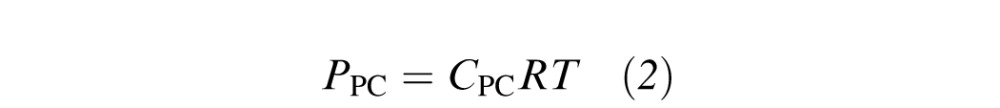
where *C*_PC_ is the molar density (or concentration) of perfluor-n-octane in vapor phase and *R* the universal gas constant.

The dependence of viscosity on temperature was determined by Mehrotra's correlation

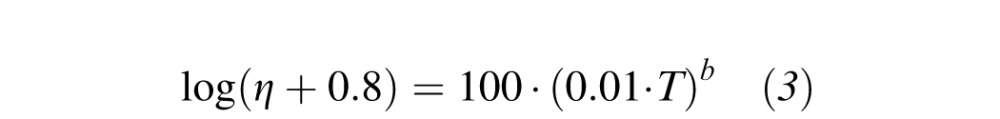
where *η* is the viscosity in mPa·s and *T* the absolute temperature. This correlation allows to predict the behavior of the viscosity as a function of temperature provided that the viscosity value at a specific temperature is known. Indeed for *T* = 298 K *η* = 5.10 mPa·s so that one obtains *b* = −4.46.

For pure liquid, if only a few experimental data are available, it is possible to estimate the temperature dependence of the vapor pressure by means of the relation of Clausius-Clapeyron

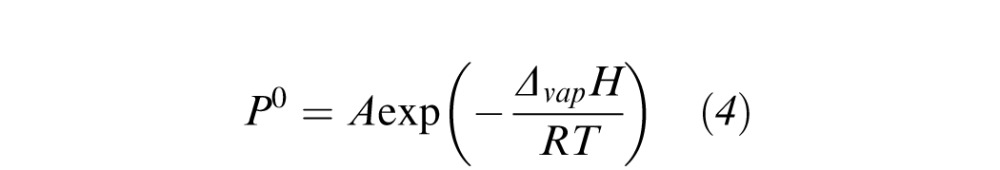
where is *A* is an unknown constant and Δ*_vap_H* is the molar enthalpy of vaporization (energy required to transform a mole of liquid into gas at a given pressure), which is linked to the entropy (Δ*_vap_S*) by the relation

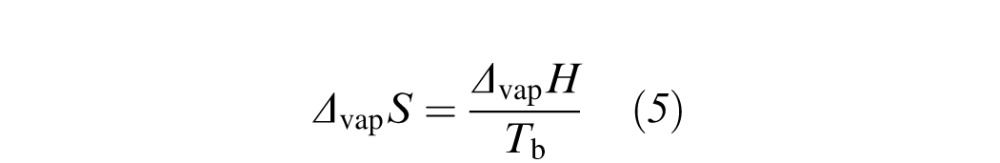
with *T*_b_ is the normal boiling point. Such a relationship is very important because the entropy can be estimated for all liquids with the Trouton's rule.

Relation between density and temperature was calculated by the coefficient of thermal expansion:

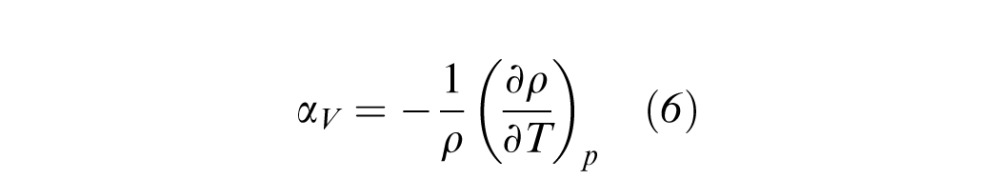
where *ρ* is the density. Because it is important to state which variables are held constant in the differentiation; the subscript P denotes the variable held constant.

Finally the dependence of viscosity on temperature was estimated by Eyring's relation:

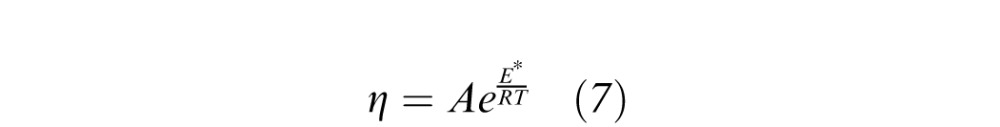
where *η* is the absolute viscosity, *A* is a constant and E^*^ is the activation energy.

## Results

**Figure 1:**
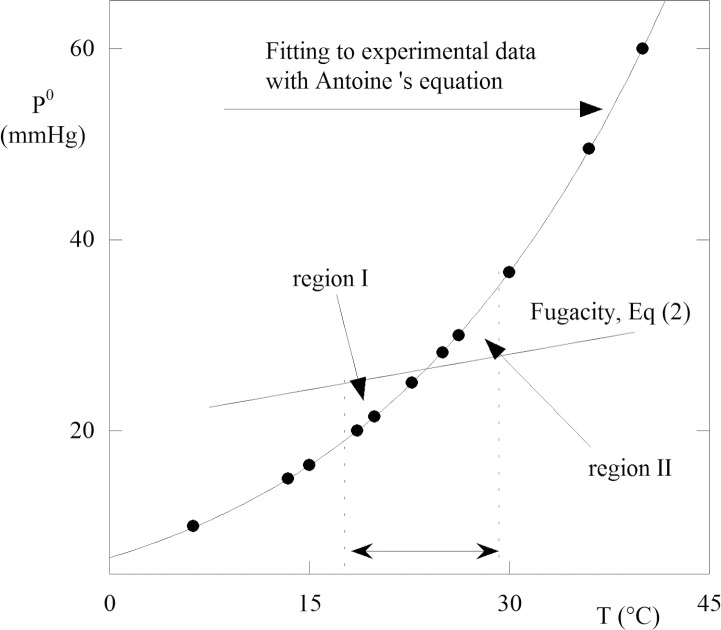
Correlation between vapor pressure and temperature for perfluorcarbon liquid. *Points* represent experimental results, the *continuous line* is the Antoine fitting to experimental data points. The *straight line* is the fugacity curve obtained supposing the vapor as an ideal gas. The *intersection*, between the *straight line* and the *liquid-vapor equilibrium curve*, in the range of temperatures surgically experienced (*T*_1_, *T*_2_), determines two regions. In the region I, the liquid has a fugacity greater than the vapor pressure then does not evaporate. In the region II, the situation is reversed. The *circle* indicates the intersection between the temperature at 28°C and the vapor pressure at 30 mm Hg. In this case the point is in region II, therefore the liquid will evaporate.

The fitting of Antoine equation (Equation 1) to experimental data of perfluor-n-octane,^22^ allows to obtain A = 18.92, B = 5892, and C = 36.7. Experimental data points and the fitting curve are plotted in Figure 1 that shows that the vapor pressure increases with temperature (i.e., decreases with increasing cohesion forces between the particles of the liquid). With increased temperature, the increasing number of particles on the surface of the liquid has a sufficient kinetic energy to overcome the cohesion forces to pass vapor state. The partial pressure (or the fugacity) of perfluor-n-octane in the gas phase, *P*_PC_, is an index showing how much the liquid–vapor system is away from equilibrium. Indeed, if we assume that the vapor phase is ideal, the partial pressure takes the form where *C*_PC_ (Equation 2) is the molar density of perfluor-n-octane in vapor phase. Generally, the vitrectomy surgery experiences a limited range of temperatures (*T*_1_, *T*_2_). Therefore, as a first approximation, one can assume the density and independent of temperature. Under this constrain, Equation 2 represents a straight line in the (*T*, *P*) plane. Using this information we may construct an operating diagram (i.e., a plot indicating whether the work condition is favorable or not to evaporation). In the (*T*, *P*) plane the segments *T* = *T*_1_ and *T* = *T*_2_ and *P*^0^(*T*) describes the curve according to Antoine's equation (Fig. 1). If the CPC value is such that Equation 2 in satisfied for *T* = *T*_Q_, which lies within the experimental range (*T*_1_, *T*_2_), then the straight line (fugacity) intersects the curve *P*^0^(*T*) at a point *Q* of the work domain. This intersection identifies two regions in the work region (Fig. 1). For points in the region I, perfluor-n-octane liquid cannot evaporates at *P*_PC_ > *P*^0^, rather the vapor condenses. On the contrary for points in the region II, *P*_PC_ < *P*^0^ and the liquid evaporates. Obviously, the intersection in the region of work depends on the slope of the line (i.e., on the molar density of perfluor-n-octane vapor). However, the knowledge of the surface properties of the liquid and the vapor-liquid equilibrium characteristics may help the surgeon to confirm the region II at the end of vitrectomy. It is worth nothing that, as far as liquids and vapors are concerned, one normally assumes that two-phase equilibria are reached almost instantaneously. This is not strictly true because superheated liquids and supercooled vapors do occur in nature–evolution to the equilibrium state, requiring nucleation of the second phase. However, the assumption is a reasonable because the evolution toward the equilibrium state is very fast when nucleation occurs.

**Figure 2:**
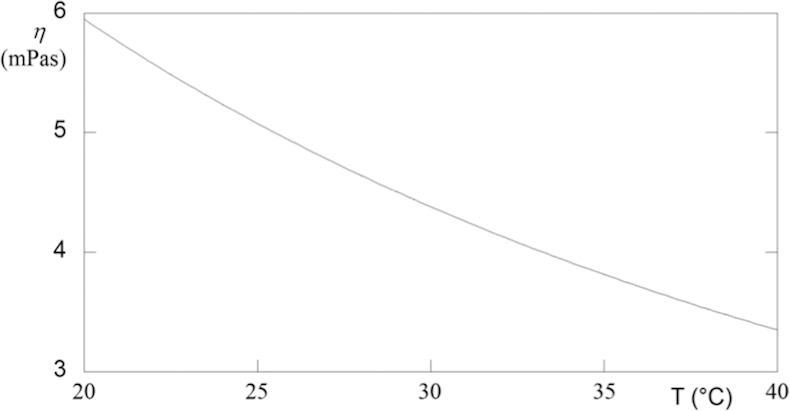
Viscosity versus temperature for perfluorcarbon liquid. The curve was obtained using experimental data from ref. 23 and applying the correlation of Mehrotra.

The viscosity of PCFL is another important property that plays a basic role in removing PCFL from eyes. However, only a few studies on this topic are available. Therefore, we determined the relationship between viscosity and temperature using the Mehrotra correlation,^23^ (Equation 3), which was tested for several hundred hydrocarbons. The results are plotted in Figure 2, in which the viscosity exhibits a remarkable fall in the range of 20°C to 40°C of the temperature. Based on Figure 2, we estimates that a change of temperature from 25°C to 40°C causes a decrease in viscosity of approximately 36%. Among all PFCLs, perfluor-n-octane has the highest vapor pressure at room temperature, which allows more complete removal of perfluor-n-octane in an open system during fluid-air exchange.

Regarding the silicone oil (SO), temperature control in the vitreous cavity can also induce changes in SV of SO. In addition, temperature control can modulate the interaction between heavy silicone oil (HSO) and PFCL responsible for the sticky oil formation.^16^ For pure liquid, although few experimental data are available, it is possible to estimate the temperature dependence of the vapor pressure by means of the relation of Clausius-Clapeyron (Equation 4). The Clausius-Clapeyron equation^22^ allows us to estimate the vapor pressure at another temperature, *T*, if the vapor pressure is known at certain temperature and the enthalpy of vaporization is known.

We note that the molar enthalpy of vaporization (i.e., the energy required to transform a mole of liquid into gas at a given pressure) is related to the normal boiling temperature of the liquid (Equation 5).

On the other hand, a comparable change in volume occurs when any liquid evaporates and become a gas. Hence, all liquids can be expected to have similar standard entropies of vaporization Δ*_vap_S* = 88 J K^−1^ mol^−1^. This observation is the Trouton's rule.^24–26^ For silicone oil, the boiling temperature is in the range of 220°C to 250°C, therefore, the Trouton's rule allows us to estimate Δ*_vap_H* = 41.9-44.5 kJmol^−1^.

**Figure 3:**
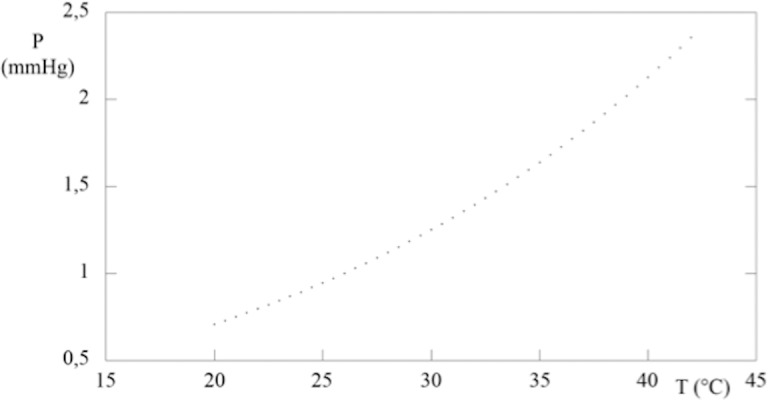
Equilibrium liquid-vapor curve, for silicone oil. The Clayperon equation was calculated making use of the Trouton's rule to evaluate the vaporization enthalpy.

As shown in Figure 3, unlike the PFCL, the pressure is always very low in the temperature range of vitreoretinal surgery, suggesting that the removal of silicone oil from eyes is more difficult as it will strongly adhere to the walls of the eye.

Generally, materials tend to expand as a function of temperature. The volume expansivity, *a*_v_, is an indication of the change in volume that occurs when the temperature changes, while the pressure remains constant. Of course, heating or cooling affects the volumetric properties of a body causing density (*ρ*) changes.^27^

Density changes with temperature may be computed by formula (Equation 6).

**Figure 4:**
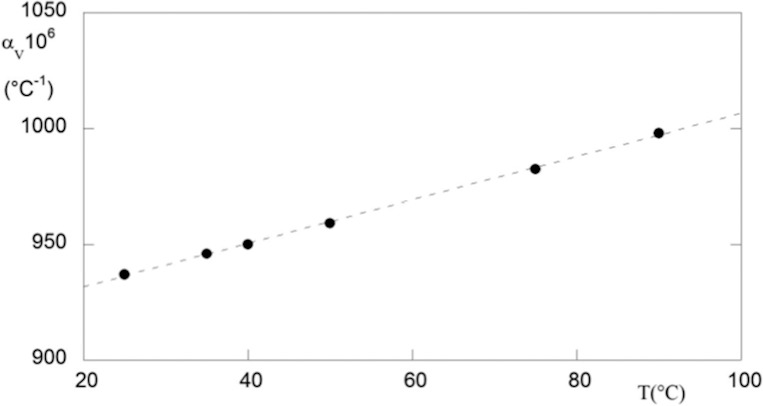
Thermal expansion versus temperature for silicone oil. The curve was evaluated directly from experimental volumetric data.

This coefficient is used to determine the rate at which the material expands as a function of temperature, which is of considerable interest to vitrectomy surgery. Indeed, various polymers of biomedical interest tend to expand and contract six to nine times more than metals.^28^ The thermal expansion develops internal stresses and stress concentrations in the polymer, which allows premature failure to occur. The thermal expansion for silicone oil was evaluated using density data previously reported.^29^ The results are plotted in Figure 4, in which the coefficient *a*_v_ increases with temperature. The principle mode of thermal energy assimilation is though increase in vibration energy of atoms. The vibrations of adjacent atoms are coupled based on the nature of atomic bonding, leading to lattice wave that transfers energy through materials. Thermal expansion results in an increase in the average distance between atoms. The extent of the phenomenon is measured by the potential energy versus interatomic spacing curve. If this curve were symmetric, there would be no net change in interatomic separation with increasing temperature and, consequently, no thermal expansion. Thus, the trend exhibits in Figure 4 is due to the asymmetric curvature of potential, rather than the increased atomic vibrational amplitude with rising temperature. Because the greater the atomic bonding energy, the deeper and narrower this potential energy, the high *a*_V_ of silicone oil values indicates that the polymer is linear or branched, because the secondary intermolecular bonds are weak, and there is a minimum of crosslinking.

Romano et al.^30^ demonstrated that it was possible to reduce the viscosity of SO using the variation of temperature, within the same interval of temperature occurring during the vitreoretinal surgery. The SV of a fluid is dependent with and inversely proportional to the changes of temperature. Using an Ubbelohde viscometer, this pilot study measured the changes in the viscosity of SO 1000 cSt and 5000 cSt according to the changes of temperature, which are typically generated during vitreoretinal surgery. The authors demonstrated that the SV tend to decrease with the increase of temperature.^30^

The surgical effects are that a decrease of SV of SO with the increase of temperature, suggesting a lesser resistance to injection removal from the vitreous cavity.

**Figure 5:**
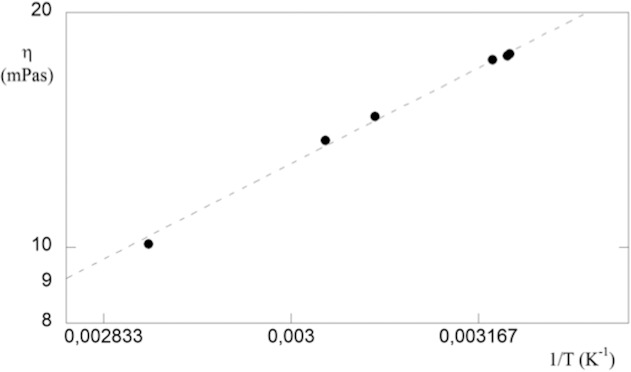
Semilog plot of dynamic viscosity versus the reciprocal of temperature for silicone. The linear fitting of experimental data proves that the Eyring model is valid. The slope of straight line is the energy barrier against flow of silicone oil.

The temperature dependence of viscosity is one of the most important variables in polymer flow. The equation commonly used to express the viscosity–temperature behavior is an Arrhenius-type equation. (Equation 7). This equation is convenient because it contains only two parameters, *A* and E^*^. Experimental data obtained from an oscillating sinker viscometer are available in the literature for this substance^29^ and are shown in the semilog plot in Figure 5. The linearity exhibited assures us of the validity of Arrhenius-like equation. The definition of E* implies the presence of a barrier against flow and for silicone oil E^*^ = (14.1 ± 0.5) kJ×mol^−1^. The advantage of this approach is that E* is independent of polymer molecular mass and distribution, as long as the molecular mass exceeds a minimum value, which closely corresponds to the entanglements molecular mass. It has been further shown that E^*^ measured at a series of constant stresses, including non-Newtonian region, gives an E^*^ identical with the value for limiting low shear viscosities.^29^

This would allow us to derive a unique functional representation for all silicones used in vitreoretinal surgery.

The dynamic viscosity and density of polydimethylsiloxane (SO) are inversely proportional to temperature. This phenomenon could be favorable for injection and extraction of the oil during surgery, due to reduction of shear stress. Therefore, the surgeon could decide to work with higher temperature, but within the physiological range of 25°C to 36°C.

Temperature control during the injection-removal of SO may reduce SV, thus the time-flow of SO throughout the sclerotomy or trocar cannula.^30^ Indeed a temperature increase from 25°C to 35°C produces a viscosity reduction of approximately 30%.

## Discussion

Increasing evidence suggests that temperature control is an important tool in vitreoretinal surgery although the relative research is still at an early stage. Temperature measurements are only possible in experiments through a vitreoretinal thermoprobe placed in the middle of vitreous cavity.

We developed a mode to describe the pressure–temperature relationship, which theoretically should indicate the ideal balance between pressure and temperature to get the PFCL vaporization in a range of temperature values that are normally reached during the vitreoretinal surgery. Measuring the temperature in vitreous cavity under air infusion allows us to control the IOP (commercially available in vitrectomy systems with the IOP controlled infusion) to obtain the vaporization of PFCL.

Regarding the interaction between PFCL and SO, the temperature also acts on sticky oil resolution. The term of “Sticky oil” describes the SO-like materials that stay glued to the inner retina at the time of SO removal.^18^

Veckeneer et al.^18^ reported in a retrospective case series such phenomena in 28 patients treated with 1000 cSt SO tamponade. The authors empathized that PFCL was used intraoperatively in the previous vitreoretinal surgery. Based on gas chromatography mass spectroscopy analysis, the authors observed a significant presence of PFCL in samples of sticky SO. In all reported cases, the authors proceeded with direct exchange between PFCL and SO 1000 cSt.

Friberg et al.^17^ described the interaction between SO and PFCL using the mass spectrometry. Particularly, the authors observed the presence of small amount of one fluid into the other over time. According to the literature, the sticky oil formation was identified in eyes previously treated with PFCL and light SO, but more when PFCL was in contact HSO.^16^

HSO is a compound formed by mixing semifluorurate (ether or alkane) to SO. According to the semifluorurate, the saturation point of HSO is the highest concentration of semifluorurate dissolved in the SO. As partially saturated fluorinated compound, HSO is more unstable than SO, and therefore more predisposed to produce emulsion and increase intraocular pressure.^31^ Adding PFCL to HSO changes the saturation point of the compound, which determines the changes of opacity and viscosity of the HSO.^16^ Such solubility equilibrium is temperature dependent. Therefore, temperature changes induce changes of the saturation of HSO by PFCL. Temperature decrease increases opalescence and viscosity of the solution.^16^ In addition, Romano et al.^16^ recently demonstrated in a laboratory study that the PFCL-HSO interactions produced a hyperviscous solution with higher SV and the characteristics of sticky oil. Without PFCL, the hyperviscous solution was not produced. Therefore, the direct exchange of PFCL-HSO is not recommended during vitreoretinal surgery because of the direct contact between PFCL-HSO at the oil interface.^32^ Sticky oil may be just defined as a hype viscous compound produced by interacting with PFCL. Whereas the stickiness of the compound may be related to a drop in the ST of the surrounding aqueous proteins mixed with PFCL.^16^ We therefore demonstrate the relation between the temperature and silicone oil changes. By increasing the temperature between physiological ΔT, the SV decreases with the resolution of the sticky oil.^16^

Temperature control may also improve the retinal-neuroprotective effects.

The fast temperature fluctuation during the vitreoretinal surgery may have effects on the functional visual recovery.^12^ This hypothesis has been confirmed in the study conducted by Horiguchi et al.,^11^ in which an abnormally delayed peak time in six eyes was associated with reduced amplitudes on electroretinograph readings after pars plana vitrectomy under infusion at room temperature.

Two groups treated with bottle infusion at room temperature (25°C) and 34°C were compared in this study. The hypothesis for electroretinogram (ERG) changes was that the temperature may inhibit the viability and the metabolic activity of retinal cells, responsible for the electrical reaction to the light. The ERG changes were reversible, however, such reversible findings may not be applied to patients with severe retinal conditions such as diabetic retinopathy with impaired macular perfusion.^12^

Avoiding temperature fluctuation during the surgery may have significant retinal-neuroprotective effects. For example, the target temperature in systemic therapeutical approaches may improve neuroprotection. Temperature control may also expand the therapeutic time window for other treatment strategies.^9^ Further studies are required to determine the target temperature according to the etiology (e.g., diabetes) and local condition of patients as well as the length of operation.

We believe that the control of the temperature is helpful in the management of intraocular compounds, and may results in safer vitreoretinal surgery.

Nowadays, there are no commercial devices available to measure and control the temperature during vitrectomy. However, based on the observation made here there are some important take home messages:

- Avoid the direct PFCL-HSO exchange, as it could induce the sticky oil formation at time of removal of HSO;
- In the presence of sticky oil perform a fluid–air exchange, as in a few minutes the air will cause the intraocular temperature to raise inducing changes in the saturation of HSO by PFCL, and this will help the resolution of sticky oil;
- Avoid cooling the infusion bottle for the vitreoretinal surgery, as this will generate more fluctuation and decrease of the intraocular temperature;
- Vaporization of residual PFCL can be achieved under air infusion of 35 mm Hg at 26.2°C; and
- Ideally, the intraocular temperature during vitrectomy with SO should be maintained at 35°C, rather than at the more commonly used room temperature of 22°C. This will also have the effect of reducing the adhesive forces between the SO and internal surface of the eye. Overall, this will facilitate a quicker and more complete oil extraction.

However, further investigations are needed to understand the applications, risks, and benefits of temperature variations and to confirm the clinical significance of these findings.

## References

[1] Frink M, Flohe S, van Griensven M, Mommsen P, Hildebrand F. Facts and fiction: the impact of hypothermia on molecular mechanisms following major challenge. *Mediators Inflamm*. 2012; 2012: 762840. 22481864 10.1155/2012/762840 PMC3316953

[2] Hildebrand F, van Griensven M, Giannoudis P, Impact of hypothermia on the immunologic response after trauma and elective surgery. *Surg Technol Int*. 2005; 14: 41–50. 16525953

[3] Howes D, Ohley W, Dorian P, Rapid induction of therapeutic hypothermia using convective-immersion surface cooling: safety, efficacy and outcomes. *Resuscitation*. 2010; 81: 388–392. 20122778 10.1016/j.resuscitation.2009.12.025 PMC2852683

[4] Motamedi GK, Lesser RP, Vicini S. Therapeutic brain hypothermia its mechanisms of action, and its prospects as a treatment for epilepsy. *Epilepsia*. 2013; 54: 959–970. 23551057 10.1111/epi.12144

[5] Polderman KH. Mechanisms of action, physiological effects, and complications of hypothermia. *Crit Care Med*. 2009; 37: S186–S202. 19535947 10.1097/CCM.0b013e3181aa5241

[6] Valeri CR, MacGregor H, Cassidy G, Tinney R, Pompei F. Effects of temperature on bleeding time and clotting time in normal male and female volunteers. *Crit Care Med*. 1995; 23: 698–704. 7712760 10.1097/00003246-199504000-00019

[7] Bouchama A, Knochel JP. Heat stroke. *New Engl J Med*. 2002; 346: 1978–1988. 12075060 10.1056/NEJMra011089

[8] Held B. Temperature effects on low-frequency electric microfields in multicomponent plasmas. *Phys Rev A*. 1985; 31: 1939–1940. 9895707 10.1103/physreva.31.1939

[9] Tamai K, Toumoto E, Majima A. Protective effects of local hypothermia in vitrectomy under fluctuating intraocular pressure. *Exp Eye Res*. 1997; 65: 733–738. 9441696 10.1006/exer.1997.0386

[10] Landers MB, III Watson JS, Ulrich JN, Quiroz-Mercado H. Determination of retinal and vitreous temperature in vitrectomy. *Retina (Philadelphia, Pa)*. 2012; 32: 172–176. 10.1097/IAE.0b013e31821c3ee0 21878844

[11] Horiguchi M, Miyake Y. Effect of temperature on electroretinograph readings during closed vitrectomy in humans. *Arch Ophthalmol*. 1991; 109: 1127–1129. 1867557 10.1001/archopht.1991.01080080087035

[12] Romano MR, Vallejo-Garcia JL, Romano V, Angi M, Vinciguerra P, Costagliola C. Thermodynamics of vitreoretinal surgery. *Curr Eye Res*. 2013; 38: 371–374. 23230955 10.3109/02713683.2012.745160

[13] Romano MR, Vallejo-Garcia JL, Castellani C, Costagliola C, Vinciguerra P. Residual perfluorocarbon liquid (PFCL) in human eyes. *Ann Acad Med Singapore*. 2014; 43: 195–196. 24714716

[14] Romano MR, Baddon C, Heimann H, Wong D, Hiscott P. Histopathological findings in an epimacular membrane after intraoperative use of perfluorocarbon liquid. *Eye (Lond)*. 2010; 24: 740–742. 19543239 10.1038/eye.2009.148

[15] de Queiroz JM, Jr Blanks JC, Ozler SA, Alfaro DV, Liggett PE. Subretinal perfluorocarbon liquids. An experimental study. *Retina (Philadelphia Pa)*. 1992; 12: S33–S39. 1455081

[16] Romano MR, Vallejo-Garcia JL, Parmeggiani F, Romano V, Vinciguerra P. Interaction between perfluorcarbon liquid and heavy silicone oil: risk factor for “sticky oil” formation. *Curr Eye Res*. 2012; 37: 563–566. 22578277 10.3109/02713683.2012.669511

[17] Friberg TR, Siska PE, Somayajula K, Williams J, Eller AW. Interactions of perfluorocarbon liquids and silicone oil as characterized by mass spectrometry. *Graefes Arch Clin Exp Ophthalmol*. 2003; 241: 809–815. 13680249 10.1007/s00417-003-0698-5

[18] Veckeneer MA, de Voogd S, Lindstedt EW, Menz DH, van Meurs JC. An epidemic of sticky silicone oil at the Rotterdam Eye Hospital. Patient review and chemical analyses. *Graefes Arch Clin Exp Ophthalmol*. 2008; 246: 917–922. 18299876 10.1007/s00417-008-0768-9

[19] Peyman GA, Schulman JA, Sullivan B. Perfluorocarbon liquids in ophthalmology. *Surv Ophthalmol*. 1995; 39: 375–395. 7604361 10.1016/s0039-6257(05)80093-1

[20] Romano MR. Re: Primary 23-gauge transconjunctival sutureless vitrectomy for rhegmatogenous retinal detachment. *Retina (Philadelphia Pa)*. 2009; 29:1547;author reply 1547-1548. 10.1097/IAE.0b013e3181b7746a 19898193

[21] Owen MJ. The surface activity of silicones: a short review. *Ind Eng Che Prod Res Dev*. 1980; 19: 97–103.

[22] Astarita G. *Thermodynamics*. New York: Plenum Press; 2004.

[23] Mehrotra AK. Correlation and prediction of the viscosity of pure hydrocarbons. *Can J Chem Eng*. 1994; 72: 554–557.

[24] Nash LK. Trouton and T-H.E rule. *J Chem Educ*. 1984; 61: 981–984.

[25] Rooney JJ. Trouton's rule. *Nature*. 1990; 348: 398. 2247145

[26] Green JA, Irudayan SJ, Henchman RH. Molecular interpretation of Trouton's and Hildebrand's rule for the entropy of vaporization of aliquid. *J Chem Thermodynamics*. 2011; 43: 868–872.

[27] Ambrosone L, Sartorio R, Vescio A, Vitagliano V. Volumetric properties of aqueous solutions of ethylen glycol oligomers at 25°C. *J Chem Soc Faraday Trans*. 1996; 92: 1163–1166.

[28] Mitchell BS. *An introduction to material engineering and science*. Hoboken NJ: Wiley-Interscience; 2004.

[29] He Y-C, Xu X-J, Yang L-J, Ding B. Viscosity modeling for ionic liquid solutions by eyring-wilson equation. *Chem Industry Chem Eng Quarterly*. 2012; 18: 441–447.

[30] Romano MR, Vinciguerra R, Vinciguerra P. Sutureless silicone oil removal: a quick and safe technique. *Retina (Philadelphia Pa)*. 2013; 33: 1090–1091. 10.1097/IAE.0b013e31828297d4 23549094

[31] Wong D, Kumar I, Quah SA, Ali H, Valldeperas X, Romano MR. Comparison of postoperative intraocular pressure in patients with Densiron-68 vs conventional silicone oil: a case-control study. *Eye (Lond)*. 2009; 23: 190–194. 18064055 10.1038/sj.eye.6703055

[32] Romano MR, Zenoni S, Arpa P, Mariotti C. Mixture of ether and silicone oil for the treatment of inferior complicated retinal detachment. *Eur J Ophthalmol*. 2013; 23: 230–235. 23138665 10.5301/ejo.5000215

